# The ribonuclease E regulator RebA is essential for diazotrophic growth in the cyanobacterium *Anabaena* PCC 7120

**DOI:** 10.1002/mlf2.70045

**Published:** 2025-10-27

**Authors:** Sujuan Liu, Zhenyu Wang, Guiming Lin, Wenkai Li, Xiaoli Zeng, Ju‐Yuan Zhang, Cheng‐Cai Zhang

**Affiliations:** ^1^ Key Laboratory of Algal Biology, Institute of Hydrobiology Chinese Academy of Sciences Wuhan China; ^2^ Institut WUT‐AMU Aix‐Marseille Université and Wuhan University of Technology Wuhan China; ^3^ University of Chinese Academy of Sciences Beijing China; ^4^ State Key Laboratory of Lake and Watershed Science for Water Security Chinese Academy of Sciences Nanjing China; ^5^ Hubei Hongshan Laboratory Wuhan China

**Keywords:** cyanobacteria, diazotrophic growth, heterocyst, ribonuclease E, RNA metabolism

## Abstract

Ribonuclease E (RNase E) is central to bacterial RNA metabolism. In cyanobacteria, its activity is inhibited by RebA, a key mechanism for controlling cell morphology. Here, we demonstrate that *rebA* is essential for diazotrophic growth of *Anabaena* PCC 7120, a filamentous cyanobacterium capable of forming heterocysts—specialized nitrogen‐fixing cells—upon nitrogen starvation. The *rebA* mutant strain (Δ*rebA*) showed severe growth defects in nitrogen‐deprived conditions, despite forming more heterocysts than the wild type. With a GFP fusion strain, we show that RebA is transiently upregulated during heterocyst differentiation. Microscopic and ultrastructural analyses revealed that Δ*rebA* heterocysts accumulated abnormally large cyanophycin granules, while vegetative cells showed reduced pigment levels and disorganized thylakoid membranes, phenotypes indicative of a severe nitrogen deficiency response. However, esculin tracer diffusion and SepJ‐GFP localization in Δ*rebA* were comparable to the wild type, suggesting that cell–cell communication via septal junctions remains functional. Thus, the growth defect likely results from impaired degradation or mobilization of fixed nitrogen. Notably, the Δ*rebA* phenotype could be rescued only by wild‐type RebA, but not by variants unable to bind RNase E, indicating that RebA's function depends on its modulation of RNase E activity. Together, these findings reveal a key posttranscriptional mechanism linking RNase E regulation to heterocyst development and intercellular nutrient transfer, highlighting the importance of regulated RNA metabolism for diazotrophic growth.

## Impact statement

Cyanobacteria play a vital role in global nitrogen cycling and hold great potential for applications in sustainable agriculture and biotechnology. This study uncovers a previously unrecognized regulatory mechanism in cyanobacterial nitrogen fixation, demonstrating that RebA, an inhibitor of ribonuclease E (RNase E), is essential for heterocyst differentiation and function in *Anabaena* PCC 7120. By regulating RNase E activity, RebA influences heterocyst frequency, thylakoid integrity, pigment synthesis, and fixed nitrogen transport. These findings provide novel insights into the role of RNA metabolism in diazotrophic growth, advancing our understanding of cyanobacterial physiology and its broader ecological and biotechnological implications.

## INTRODUCTION

Ribonuclease E (RNase E) is a central regulator in bacterial RNA metabolism. As an endoribonuclease, it initiates the decay of messenger RNAs (mRNAs), ribosomal RNAs (rRNAs), and small noncoding RNAs (sRNAs), playing a crucial role in RNA homeostasis and gene regulation^1^, ^2^, ^3^. By controlling RNA turnover, RNase E enables bacteria to rapidly adjust gene expression in response to environmental changes.

RNase E homologs are widely distributed across bacterial phyla, including *Proteobacteria*, *Actinobacteria*, *Bacteroidetes*, *Chlamydiae*, *Cyanobacteria*, and *Firmicutes* ^4^. These homologs share a well‐conserved N‐terminal catalytic region and a highly variable C‐terminal region, the latter often serving as a scaffold for RNA degradosome assembly. In *Escherichia coli*, this degradosome includes RNase E, PNPase, RhlB helicase, and enolase^5^, ^6^, ^7^. In other species, such as *Rhodobacter capsulatus* and *Caulobacter crescentus*, the complex has a distinct composition^8^, ^9^, underscoring the evolutionary flexibility of RNase E‐based RNA degradosome.

Given the global influence of RNase E on RNA metabolism, its activity must be tightly controlled. To date, several mechanisms have been identified in a limited number of bacterial species. For example, in *E. coli*, regulatory proteins, such as RraA and the ribosomal protein L4, modulate RNase E by interacting with its noncatalytic C‐terminal region^10^, ^11^, ^12^. Viral inhibitors, such as Dip from phage ϕKZ, also target this region to suppress host RNase E activity^13^.

Cyanobacteria are distinguished by their ability to perform oxygenic photosynthesis, setting them apart phylogenetically from other bacterial phyla. RNase E is essential for viability in different cyanobacterial species^14^, ^15^, ^16^. In the filamentous cyanobacterium *Anabaena* PCC 7120 (*Anabaena* hereafter), RNase E interacts with PNPase, RNase II, and the RNA helicase CrhB, forming an RNA degradosome distinct from those found in other bacteria^17^, ^18^, ^19^. Recently, we identified RebA, a conserved cyanobacterial protein, as a negative regulator of RNase E activity in *Anabaena* ^16^. In contrast to known RNase E regulators that bind the C‐terminal region, RebA interacts with the 5′‐sensor domain in the N‐terminal catalytic region of RNase E, impairing RNase E's ability to bind RNA substrates and thereby inhibiting cleavage. Altering RebA levels affects cell morphology, highlighting its physiological importance in regulating RNase E activity.

*Anabaena* shows a remarkable form of cellular differentiation in response to nitrogen starvation, forming heterocysts—specialized cells that fix atmospheric nitrogen. These heterocysts are spaced semi‐regularly along the filament and develop thick cell envelopes to protect nitrogenase from oxygen^20^, ^21^. Fixed nitrogen is stored at the polar region of the heterocyst as cyanophycin granules (also known as polar granules), which are later degraded into dipeptides and transferred to vegetative cells via septal junctions^22^. Heterocyst differentiation is a tightly regulated process involving coordinated gene expression and key regulatory proteins, such as HetR, PatS, HetN, HetF, and PatU3^23^, ^24^, ^25^.

While the transcriptional regulation of heterocyst development is well characterized, the role of posttranscriptional mechanisms—particularly RNA metabolism—remains poorly understood. Here, we demonstrate that RebA‐mediated inhibition of RNase E is essential for proper heterocyst differentiation and function. Our findings uncover a critical link between regulated RNA metabolism and heterocyst development in *Anabaena*, providing new insights into the posttranscriptional control of diazotrophic growth.

## RESULTS

### The *rebA* gene is essential for diazotrophic growth of *Anabaena*

**Figure 1:**
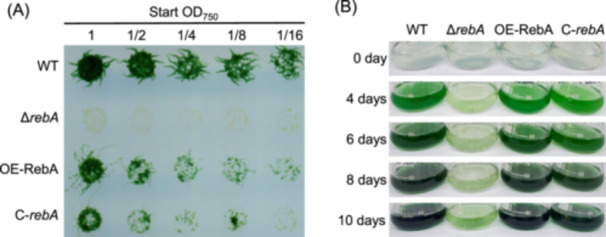
Effect of RebA levels on diazotrophic growth. (A) Growth of the wild type (WT), *rebA* deletion mutant (Δ*rebA*), RebA overexpression strain (OE‐RebA), and Δ*rebA*‐complemented strain (C‐*rebA*) on a BG11_0_ agar plate. Fresh cultures grown in liquid BG11 medium (containing nitrate) were washed with BG11_0_ medium (lacking combined nitrogen), adjusted to an initial OD_750_ of 1.0, and then serially diluted. The diluted cultures were spotted onto the plate, which was photographed after 14 days of incubation. (B) Growth of the same strains in liquid BG11_0_ medium. The initial OD_750_ of each strain was 0.05. The culture flasks were photographed at indicated time points after nitrogen depletion.

To assess the role of RebA in diazotrophic growth, we examined the growth of the *rebA* deletion mutant (Δ*rebA*), the complemented strain (C‐*rebA*), the RebA overexpression strain (OE‐RebA), and the wild type (WT) in BG11_0_ medium, which lacks combined nitrogen. On solid BG11_0_ plates, the WT displayed robust growth, while Δ*rebA* showed a complete loss of viability across all inoculum concentrations (Figure 1A). OE‐RebA and C‐*rebA* were able to grow, though more slowly than the WT. Similar trends were observed in liquid BG11_0_ cultures: WT, OE‐RebA, and C‐*rebA* showed comparable growth over time, whereas Δ*rebA* showed significantly slower growth and remained pale even after 10 days (Figure 1B). Together, these results demonstrate that RebA is essential for diazotrophic growth in *Anabaena*.

### Deletion of *rebA* increases heterocyst frequency

**Figure 2:**
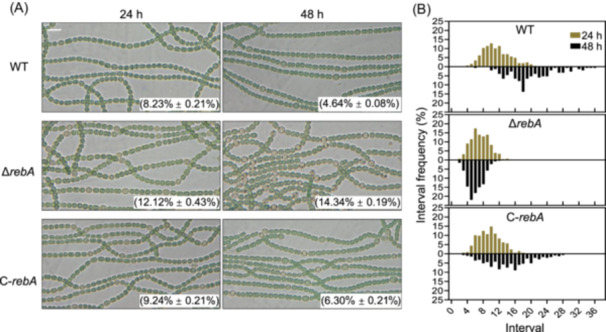
Heterocyst differentiation in the Δ*rebA* strain. (A) Microscopic images of WT, Δ*rebA*, and C‐*rebA* filaments after 24 and 48 h of nitrogen deprivation. Heterocyst frequency for each sample is indicated. Values are presented as mean ± standard deviation, based on three independent biological experiments, each with at least 2000 analyzed cells per sample. Scale bar, 10 μm. (B) Distribution of heterocyst intervals along filaments in WT, Δ*rebA*, and C‐*rebA* strains at 24 and 48 h after nitrogen depletion. The images used for interval measurements are the same as those used for heterocyst frequency calculations in (A).

Given that Δ*rebA* showed poor growth under combined‐nitrogen deprivation, we first examined its ability to differentiate heterocysts. We grew Δ*rebA*, C‐*rebA,* and WT under nitrogen‐deprived conditions for 24 and 48 h and observed their filaments under a microscope. Surprisingly, Δ*rebA* was able to differentiate heterocysts at a much higher frequency than WT and C‐*rebA* (Figure 2A). After 24 h of nitrogen deprivation, heterocyst frequency in Δ*rebA* was 12.12%, compared to 8.23% in WT and 9.24% in C‐*rebA*. After 48 h, the heterocyst frequency in Δ*rebA* increased to 14.34%, while WT and C‐*rebA* showed a decrease to 4.64% and 6.30%, respectively. Notably, many Δ*rebA* heterocysts contained large cyanophycin granules after 48 h (Figure 2A), suggesting active nitrogen fixation.

We also analyzed heterocyst spacing along the filaments. After 24 h, WT and C‐*rebA* filaments displayed heterocyst intervals of mostly 8–14 and 6–13 vegetative cells, respectively, while Δ*rebA* filaments had reduced intervals of only 4–9 cells. By 48 h, WT and C‐*rebA* strains showed increased heterocyst intervals, consistent with a decrease in heterocyst frequency, whereas Δ*rebA* continued to show further decrease in vegetative cell spacing (Figure 2B). These findings suggest that RebA plays an inhibitory role in heterocyst differentiation, maintaining normal frequency and spacing.

### RebA is upregulated during heterocyst differentiation

**Figure 3:**
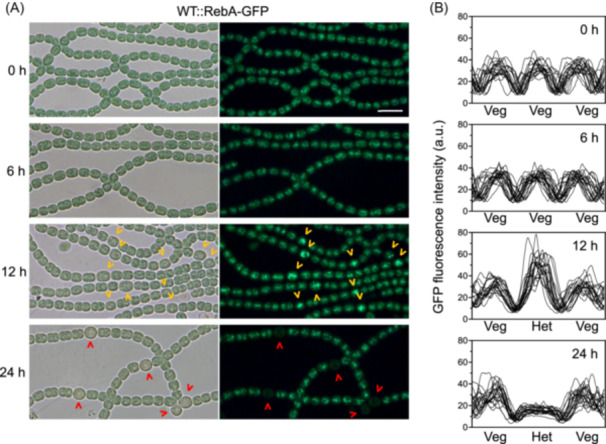
Expression of RebA in vegetative cells and differentiating cells after nitrogen deprivation. (A) Microscopic images of WT::RebA‐GFP filaments grown under nitrogen‐deprived conditions for the indicated time periods. Bright field (left) and GFP fluorescence (right) are shown. Identical microscope settings were used for fluorescent microscopy. Orange and red arrowheads indicate proheterocysts and mature heterocysts, respectively. Scale bar, 10 µm. (B) Quantification of GFP fluorescence along the filaments after nitrogen depletion. At each time point, the fluorescence intensity was measured in 20 representative short filaments, each consisting of three vegetative cells (Veg) or one proheterocyst (or mature heterocyst) (Het) with two surrounding vegetative cells. Fluorescence intensities are plotted in arbitrary units (a.u.).

To explore how RebA is involved in the process of heterocyst development, we tracked its expression using a RebA–GFP fusion strain. Under nitrogen‐replete conditions and up to 6 h after nitrogen deprivation, RebA–GFP fluorescence was uniformly distributed along the filaments. However, at 12 h, fluorescence became significantly enriched in differentiating cells (proheterocysts), with no noticeable fluorescence change in vegetative cells (Figure 3A,B). By 24 h, RebA–GFP fluorescence was nearly undetectable in mature heterocysts. These observations suggest that RebA is transiently upregulated during mid‐stage differentiation and downregulated upon maturation, reinforcing its role in heterocyst formation and function.

### RebA functions downstream of HetR in the regulatory cascade

**Figure 4:**
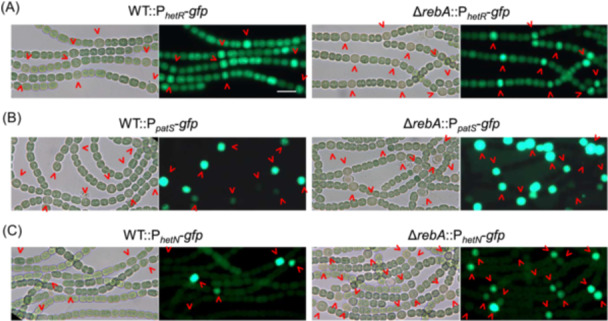
Expression of *hetR*, *patS*, and *hetN* in the Δ*rebA* strain. Bright‐field and GFP fluorescence images of the WT and Δ*rebA* strains carrying the *gfp* gene under the control of the *hetR* promoter (P_*hetR*_‐*gfp*) (A), the *patS* promoter (P_*patS*_‐*gfp*) (B), and the *hetN* promoter (P_*hetN*_‐*gfp*) (C) are shown. Images are of cultures deprived of combined nitrogen for 24 h. Red arrowheads indicate proheterocysts or mature heterocysts. Scale bar, 10 μm.

Heterocyst differentiation is a complex, multi‐gene‐regulated process, with the transcription factor HetR being the master positive regulator^24^. The activity of HetR is repressed by PatS and HetN. To determine if RebA affects this regulatory cascade, we analyzed the expression of *hetR*, *patS*, and *hetN* using GFP transcriptional fusions in both WT and Δ*rebA* backgrounds. After 24 h of nitrogen deprivation, the expression of *hetR* and *hetN* was regulated similarly in the WT and Δ*rebA*, showing strong upregulation in heterocysts (Figure 4A,C). However, *patS*, which is normally restricted to heterocysts in WT, was also upregulated in many vegetative cells of Δ*rebA* (Figure 4B). Moreover, heterocyst formation in Δ*rebA* was completely blocked by overexpressing *patS* or *hetN* (Figure S1). These results suggest that RebA prevents excessive differentiation by acting downstream of HetR, and it also suppresses *patS* expression in vegetative cells.

### Δ*rebA* heterocysts are functional in nitrogen fixation

Since Δ*rebA* formed numerous heterocysts and yet failed to grow diazotrophically, we assessed its nitrogen‐fixing efficiency. Using the acetylene reduction assays, we measured the nitrogenase activity in WT and Δ*rebA* cultures under nitrogen‐deprived conditions in both oxic and anoxic environments (Table 1). After 24 h of nitrogen deprivation, WT showed nitrogenase activities of 177.25 and 211.25 nmol C_2_H_4_ mg protein^−1^ h^−1^ under oxic and anoxic conditions, respectively. By 48 h, these values slightly decreased to 161.25 and 175.50 nmol C_2_H_4_ mg protein^−1^ h^−1^, respectively. Δ*rebA* displayed similar nitrogenase activities: 185.75 and 205.25 nmol C_2_H_4_ mg protein^−1^ h^−1^ after 24 h, and 158.25 and 174.25 nmol C_2_H_4_ mg protein^−1^ h^−1^ after 48 h. These results indicate that the inability of Δ*rebA* to support diazotrophic growth is not due to impaired nitrogen fixation. Furthermore, since Δ*rebA* showed nitrogenase activity comparable to WT under both oxic and anoxic conditions, its heterocysts likely have an intact envelope structure capable of maintaining the microoxic environment necessary for nitrogenase function.

**Table 1:** Nitrogenase activity of WT and Δ*rebA* under oxic and anoxic conditions.

Strain	Nitrogenase activity (nmol C_2_H_4_ mg protein^−1^ h^−1^)			
	Oxic		Anoxic	
	24 h	48 h	24 h	48 h
WT	177.25 ± 8.02	161.25 ± 0.51	211.25 ± 3.25	175.50 ± 0.52
Δ*rebA*	185.75 ± 0.25	158.25 ± 0.41	205.25 ± 4.07	174.25 ± 1.03

Nitrogenase activities were measured using the acetylene reduction assay after 24 and 48 h of nitrogen deprivation. Values represent mean ± standard deviations (*n* = 3 biological replicates).

### Δ*rebA* does not seem to affect septal junction function

**Figure 5:**
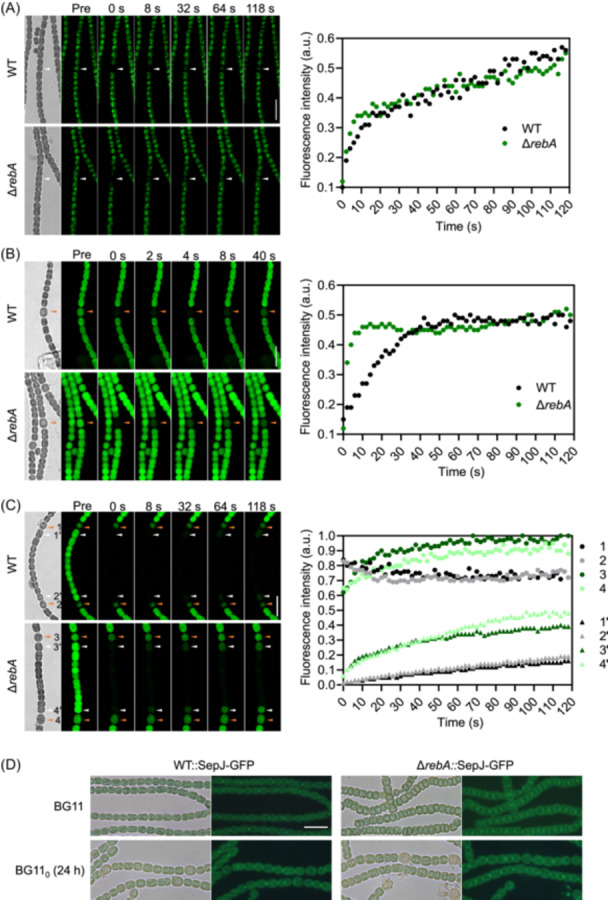
Esculin diffusion between neighboring cells and subcellular localization of SepJ in Δ*rebA*. (A) Esculin transfer between vegetative cells. WT and Δ*rebA* strains were cultured in BG11 medium and loaded with esculin. Representative FRAP time‐lapse images show esculin fluorescence (pseudocolored in green) distribution before bleaching (pre), immediately after bleaching (0 s), and at other indicated time points. White arrowheads denote the bleached cells. The right panel shows the fluorescence recovery curves of the bleached vegetative cell (measured at 2 s intervals over 120 s). Fluorescence intensity is normalized to the a.u., where 1 a.u. = 100% recovery. (B) Esculin transfer from vegetative cell to heterocyst. WT and Δ*rebA* mutant strains cultured in BG11 medium were transferred into BG11_0_ medium for 24 h before esculin labeling. The right panel displays fluorescence recovery kinetics in photobleached heterocysts. (C) Esculin transfer from heterocyst to vegetative cells. Under identical culture conditions as those in panel (B), fluorescence dynamics were simultaneously monitored in both heterocysts (designated 1–4; orange arrowheads) and adjacent vegetative cells (designated 1′–4′; white arrowheads) following photobleaching of all intervening vegetative cells between two heterocysts. The reduced heterocyst fluorescence at *t* = 0 suggests that esculin diffused out during bleaching. (D) Subcellular localization of SepJ in WT::SepJ‐GFP and Δ*rebA*::SepJ‐GFP strains. Scale bars = 10 μm (A–D).

Although heterocysts in Δ*rebA* had a nitrogenase activity comparable to that of the WT, they grew very slowly in BG11_0_ liquid medium. One possibility is that the fixed nitrogen could not be transported efficiently along the filament. In filamentous cyanobacteria, the intercellular exchange of the nutrients is mainly through the septal junctions, which are structures that connect the cytoplasms of neighboring cells^26^, ^27^. To determine whether RebA plays a role in the function of septal junctions, we first checked if the intercellular diffusion of the small fluorescent tracer esculin was affected in Δ*rebA*. Esculin is a 340‐Da fluorescent sucrose analog that can be efficiently imported into the cytoplasm of *Anabaena* cells and diffuse through septal junctions^27^. The filaments were first loaded with esculin, and then the fluorescence recovery after photobleaching (FRAP) assay was applied to determine the efficiency of esculin diffusion between neighboring cells. Similar assays have been widely used to characterize molecular exchange through septal junctions in *Anabaena* ^28^, ^29^. We first assessed esculin transfer between vegetative cells in the filaments grown in BG11 medium. The results showed that bleached WT and Δ*rebA* cells showed similar fluorescence recovery rates, indicating that esculin transfer between vegetative cells in Δ*rebA* is not affected (Figure 5A). Using filaments grown in BG11₀ for 24 h, we additionally assessed esculin transfer between vegetative cells and heterocysts. The results showed that both vegetative cell‐to‐heterocyst and heterocyst‐to‐vegetative cell esculin transfer in the Δ*rebA* mutant even displayed a slightly higher speed than in WT (Figure 5B,C).

One of the septal proteins, SepJ, is well known to be critical for the function of septal junctions^30^, ^31^, ^32^. We also compared the subcellular localization of SepJ in Δ*rebA* and WT, with the GFP translational fusion strains. SepJ‐GFP was found to be correctly localized at the septa in both strains, regardless of the culture conditions tested (Figure 5D). Together, these results suggest that the Δ*rebA* strain retains functional septal junctions.

### Δ*rebA* has reduced photosynthetic pigments

**Figure 6:**
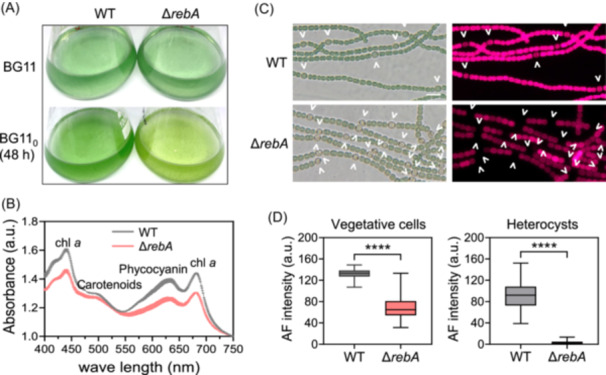
Photosynthetic pigment levels in Δ*rebA* after nitrogen deprivation. (A) Cultures of WT and Δ*rebA* in BG11 and BG11_0_ for 48 h. (B) Whole‐cell absorption spectra of exponentially grown WT (gray) and Δ*rebA* (red) cells. Spectra were measured from three biological replicates. chl *a*, chlorophyll *a*. (C) Bright‐field (left) and autofluorescence (right) images of the WT and Δ*rebA* filaments after 48 h of nitrogen deprivation. The exposure time for each sample was 10 ms. White arrows indicate heterocysts. Scale bar, 10 μm. (D) Quantification of autofluorescence (AF) intensity in vegetative cells and heterocysts from (C). At least 200 vegetative cells and 50 heterocysts were analyzed using ImageJ. Fluorescence intensities are expressed in a.u. Boxplot visualization and statistical analysis were performed using GraphPad Prism software. Each boxplot includes the interquartile range (25th to 75th percentile), with the black line representing the median value. Sample differences were evaluated using a paired Student's *t*‐test, revealing highly significant differences (****p < 0.0001).

After 48 h of nitrogen deprivation, Δ*rebA* cultures showed a noticeable yellowish appearance compared to WT (Figure 6A), suggesting a reduction in photosynthetic pigments. To quantify this difference, we measured the whole‐cell absorption spectra of Δ*rebA* and WT cells. Compared to WT, Δ*rebA* cells had significantly lower peaks corresponding to chlorophyll *a* and phycobiliproteins (Figure 6B). Fluorescence microscopy further confirmed these differences. In WT filaments, vegetative cells showed strong autofluorescence, while heterocysts appeared dimmer due to photosystem II degradation (Figure 6C). In contrast, both vegetative cells and heterocysts of Δ*rebA* displayed substantially reduced autofluorescence, with heterocysts nearly devoid of a detectable signal (Figure 6C,D). These findings indicate that the absence of RebA leads to a marked decrease in pigment content, which may reflect a nitrogen deficiency response in Δ*rebA* cells under nitrogen‐deprived conditions.

### Deletion of *rebA* leads to altered thylakoid membrane organization

Since photosynthetic pigments are primarily associated with thylakoid membranes, we further investigated whether the *rebA* deletion affected the thylakoid structure. The ultrastructures of vegetative cells and heterocysts from both the WT and Δ*rebA* strains grown in BG11_0_ for 48 h were observed using transmission electron microscopy (TEM).

**Figure 7:**
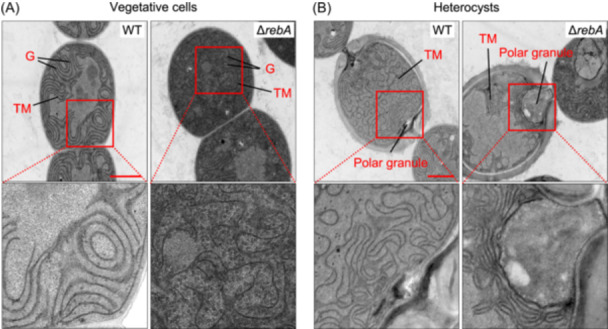
Effect of RebA levels on the ultrastructure of vegetative cells and heterocysts. (A) Transmission electron microscopy (TEM) images of vegetative cells from WT and Δ*rebA* strains after 48 h of nitrogen deprivation. Red rectangles indicate the zoomed‐in regions shown below. (B) TEM images of heterocysts in WT and Δ*rebA* strains. Glycogen granules (G), thylakoid membranes (TM), and polar cyanophycin granules are indicated. Scale bar = 1 µm.

In WT vegetative cells, the thylakoid membranes were well organized in parallel stacks, with occasional glycogen granules interspersed between the membrane layers. In contrast, Δ*rebA* vegetative cells showed highly disordered thylakoid membranes, with irregular stacking and an increased accumulation of glycogen granules (Figure 7A).

In WT heterocysts, characteristic ultrastructural features such as the polysaccharide and glycolipid layers outside of the cytoplasmic membrane, polar cyanophycin granules, and highly contorted thylakoid membranes near the cell poles were observed (Figure 7B). In Δ*rebA* heterocysts, the polysaccharide and glycolipid layers were similar to those in WT, but cyanophycin granules were abnormally large (Figure 7B), consistent with the large polar granules previously observed under the bright‐field microscope (Figure 2A). In addition, the thylakoid membranes in Δ*rebA* heterocysts were more compactly arranged near the poles but completely absent in a large region of the cytoplasm (Figure 7B).

These structural abnormalities indicate that RebA is required for maintaining thylakoid organization, which may underlie the reduced photosynthetic pigment levels observed under nitrogen‐deprived conditions.

### The interaction between RebA and RNase E is required for diazotrophic growth

**Figure 8:**
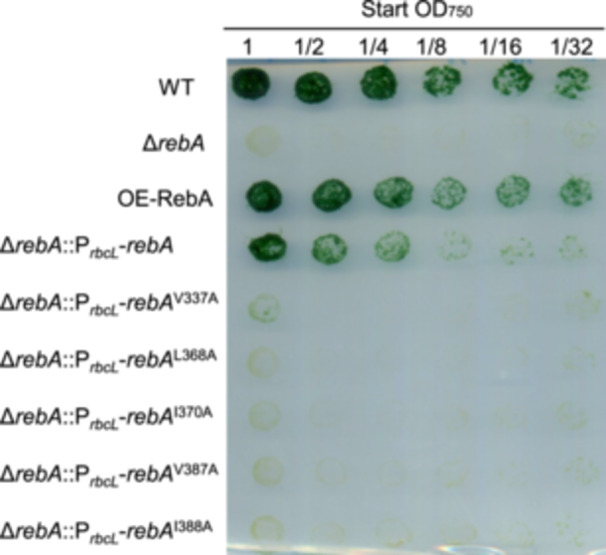
Effect of RebA–RNase E interaction on diazotrophic growth. The growth of WT, Δ*rebA*, OE‐RebA, and the Δ*rebA* strains complemented with RebA or its variants was assessed on a BG11_0_ agar plate. The experimental procedure was the same as that described in Figure 1A.

Since RebA regulates RNase E, we investigated whether its role in heterocyst differentiation depends on its ability to modulate RNase E activity. The Δ*rebA* strain was complemented with either wild‐type RebA or mutant variants unable to bind RNase E, and the growth of the resulting strains was assessed on BG11_0_ agar plates. Only the strain complemented with wild‐type RebA was able to grow, whereas those complemented with RebA variants defective in RNase E binding failed to survive under nitrogen‐deprived conditions (Figure 8). These results indicate that the regulatory activity of RebA on RNase E—rather than the presence of RebA itself—is essential for *Anabaena* growth during nitrogen starvation.

## DISCUSSION

In bacteria, the RNase E plays a pivotal role in RNA processing, turnover, and RNA‐mediated regulation. We recently demonstrated that the conserved protein RebA inhibits RNase E activity in cyanobacteria, ensuring that its broad enzymatic activity is properly controlled^16^. In both the filamentous cyanobacterium *Anabaena* and the unicellular cyanobacterium *Synechococcus* PCC 7942, RebA is essential for maintaining standard cell morphology through the modulation of RNase E activity^16^. This study further reveals the critical roles of RebA and RNase E in diazotrophic growth. The *rebA* deletion mutant Δ*rebA* formed an increased proportion of heterocysts capable of nitrogen fixation and cyanophycin accumulation, and yet, these heterocysts failed to support normal diazotrophic growth. The specific upregulation of RebA in differentiating heterocysts around 12 h after nitrogen deprivation suggests a key regulatory role during later‐stage differentiation. Furthermore, RebA variants unable to interact with RNase E failed to complement the *rebA* mutant, indicating that RebA functions through the modulation of RNase E activity. These findings expand our understanding of the regulatory roles of RebA and RNase E, underscoring the importance of regulated RNA metabolism in heterocyst differentiation and function.

In BG11_0_ medium, Δ*rebA* vegetative cells showed excessive glycogen accumulation, indicative of a severe nitrogen deficiency response^33^. These cells also showed markedly reduced photosynthetic pigment levels and disorganized thylakoid membranes—phenotypes reminiscent of cell bleaching and thylakoid degradation observed in nitrogen‐starved *Synechocystis* PCC 6803^34^. Together, these observations indicate that Δ*rebA* vegetative cells encounter strong nitrogen deficiency. At the same time, heterocysts in Δ*rebA* contained abnormally large cyanophycin granules, which may reflect impaired mobilization of fixed nitrogen. These findings suggest that the RebA‐regulated RNase E activity is required for efficient transport of fixed nitrogen from heterocysts cells to vegetative cells—an essential process for maintaining nitrogen–carbon homeostasis during diazotrophic growth.

Septal junctions, proteinaceous channels located at the cell septa, are critical for the intercellular exchange of small molecules in filamentous cyanobacteria. Their dysfunction can impede molecular transfer between cells. However, Δ*rebA* showed normal SepJ expression and localization, and the exchange of the small molecule tracer esculin between vegetative cells was unaffected (Figure 5). This suggests that septal junction functionality is not compromised in Δ*rebA*. Therefore, the inability of transporting fixed nitrogen from heterocysts to vegetative cells may instead result from abnormal cyanophycin metabolism. Inefficient degradation of cyanophycin into the transportable dipeptide β‐aspartyl‐arginine could lead to excessive accumulation at heterocyst poles, explaining the enlarged polar granules observed in Δ*rebA*. Heterocyst differentiation involves extensive transcriptional and posttranscriptional regulation^23^, ^35^. Although ribonucleases are expected to influence both general RNA degradation and specific regulatory pathways, their roles in heterocyst development remain poorly understood. Recently, we reported that the DEAD‐box RNA helicase CrhB was upregulated in differentiating cells and its depletion led to delayed heterocyst formation^36^. Here, we additionally show that RebA‐mediated regulation of RNase E activity is crucial for heterocyst differentiation and function. Since RebA is an inhibitor of RNase E, its absence is likely to result in elevated RNase E activity in Δ*rebA*. Interestingly, the absence of RebA did not affect *hetR* expression, and overexpression of HetR inhibitors (i.e., *patS* and *hetN*) suppressed heterocyst differentiation in Δ*rebA*, suggesting that RNase E activity functions downstream of HetR in the regulatory cascade. Consistently, RebA expression was upregulated in the mid‐phase of heterocyst differentiation. The increased heterocyst frequency and reduced heterocyst spacing observed in Δ*rebA* are unlikely to result from impaired intercellular diffusion of the inhibitory peptide PatS, as the intercellular exchange of small molecules appeared normal in Δ*rebA* (Figure 5). Instead, RebA may transiently inhibit RNase E activity, thereby stabilizing specific RNA molecules that regulate heterocyst frequency. Although RebA has decreased levels in mature heterocysts, its residual presence continues to modulate RNase E activity, which remains essential for heterocyst function. Future transcriptomic analyses could help identify specific RNA targets affected by RebA regulation in vegetative cells and heterocysts, further elucidating the underlying molecular mechanisms involved.

In summary, our study identifies RebA as a key regulator of heterocyst differentiation and function in *Anabaena* PCC 7120. By modulating RNase E activity, RebA ensures proper heterocyst spacing and efficient intercellular transfer of fixed nitrogen, thereby supporting diazotrophic growth. These findings highlight the essential role of posttranscriptional regulation in cyanobacterial multicellularity and reinforce the intricate connection between RNA metabolism and cellular differentiation. Future research will focus on identifying specific RNase E‐dependent pathways influenced by RebA, providing deeper insights into the regulatory mechanisms underlying diazotrophic growth in filamentous cyanobacteria.

## MATERIALS AND METHODS

### Strains and culture conditions

All the strains used in this study are listed in Table S1. Wild‐type *Anabaena* PCC 7120 and its derived mutant strains were grown photoautotrophically in BG11 medium containing nitrate or in BG11_0_ medium lacking nitrate at 30°C under light (30 μmol m^−2^s^−1^ from LED lamps). Cultures were grown in shaken liquid medium (180 rpm) or on solid medium supplemented with 1.2% (w/v) agar. When necessary, antibiotics were added at the following concentrations: neomycin (50 µg/ml), spectinomycin (5 µg/ml), or streptomycin (2.5 µg/ml). For medium transfer, cells were pelleted by centrifugation at 3000*g*, followed by three washes with the appropriate medium.

To induce gene expression via the artificial CT (copper‐ and theophylline‐responsive) promoter^18^, cells were cultivated to the logarithmic growth phase in copper‐free BG11 and then transferred to the induction medium (BG11 supplemented with 1–3 μM copper and 2 mM theophylline). For genes under the *petE* promoter, induction was performed similarly, using copper as the sole inducer.

### Construction of cyanobacterial strains

Translational fusion strains of *rebA* and *sepJ* were generated using CRISPR/Cpf1‐based genome editing as described previously^37^. Overexpression and transcriptional fusion strains of *hetR*, *patS,* and *hetN* were generated by introducing the corresponding plasmids into either wild‐type *Anabaena* or the *rebA* mutant strain via conjugation^38^, ^39^. The genotypes of all obtained strains were verified by PCR. The plasmids and the oligonucleotides used in this study are detailed in Tables S2 and S3, respectively.

### Microscopy

Bright‐field images were captured using an Sdptop EX30 microscope, while fluorescence imaging was performed using an Sdptop EX40 epifluorescence microscope. GFP fluorescence was imaged using a filter set (EX470–490, DM495LP, EM500–520) with an exposure time of 1 s. Autofluorescence of *Anabaena* cells was imaged using a filter set (EX540–580, DM585LP, EM600–650) with a 10 ms exposure time. Fluorescence images were acquired with a 100×/1.28 oil immersion lens. Cells for microscopy were harvested during the exponential growth phase (OD_750_ = 0.3–0.5) unless otherwise specified. Image analysis was conducted using ImageJ software.

### Spectrometric measurements

Wild‐type *Anabaena* and Δ*rebA* mutant strains were pre‐cultured in BG11 medium before being transferred to fresh BG11_0_ liquid medium. After 48 h of growth in BG11_0_, cells were adjusted to OD_750_ = 0.5. Whole‐cell absorption spectra were measured at room temperature using a UV‐5200 spectrophotometer (Shanghai Yuan Xi) and normalized to the absorption values at 750 nm.

### TEM

Cells were fixed with 2.5% glutaraldehyde at room temperature and subsequently post‐fixed with 1% osmium tetroxide. After dehydration through a graded ethanol series (70%–100%), samples were embedded in epoxy resin. Ultrathin sections (~70 nm) were cut and stained with uranyl acetate and lead citrate^40^. Samples were examined using a Hitachi transmission electron microscope at 80 kV.

### Nitrogenase activity assay

Nitrogenase activity was measured using an acetylene reduction assay, as previously described^41^. Briefly, filaments from BG11 medium cultures were collected, washed three times with BG11_0_ medium, and then resuspended in BG11_0_ medium. After 24 or 48 h of incubation under standard growth conditions, cell suspensions were placed in glass vials sealed with rubber stoppers and incubated in an atmosphere of 10% acetylene in air (oxic condition). For acetylene reduction in anoxic culture, vials were evacuated, flushed twice with nitrogen gas, and then sparged with nitrogen for 10–15 min before adding 10% acetylene. Additionally, 10 μM 3‐(3,4‐dichlorophenyl)‐1,1‐dimethylurea (DCMU) was added to inhibit photosynthesis. After 6 h of incubation at 30°C with shaking, ethylene (C_2_H_4_) production was measured using a Shimadzu GC‐2010 gas chromatograph equipped with a DB‐5 column and a flame ionization detector. The column temperature was set to 60°C. Activity was expressed as nmol C_2_H_4_ formed mg protein^−1^ h^−1^.

### Esculin labeling and FRAP

Staining of *Anabaena* with esculin and FRAP measurements were performed as previously described^27^, ^42^. Esculin, a fluorescent sucrose analog, is incorporated by a sucrose import system into the cytoplasm of *Anabaena* cells. For esculin labeling, *Anabaena* WT and Δ*rebA* mutant strains were cultured in BG11 medium, followed by nitrogen deprivation in BG110 medium for 24 h before esculin labeling. The filaments were then harvested, washed three times with fresh medium, resuspended in 0.5 ml of fresh medium, mixed with 10 μl of ~150 mM esculin solution, and incubated in the dark for 60 min at 30°C. Subsequently, the cells were washed, spotted onto BG110 agar‐coated glass slides (1.2%, wt/vol), and air‐dried. All measurements were carried out at room temperature (~25°C). For assays of intercellular transfer, samples were visualized using an inverted confocal laser scanning microscope (Leica DLS SP8) with a 63× oil immersion objective. Fluorescence was excited at 405 nm, and emission was monitored across a window of 425 to 485 nm with a 100 µm confocal pinhole. The pre‐bleach images were recorded using 10% intensity of a 50‐nW laser power; regions of interest were bleached at 50% laser power. Fluorescence recovery was monitored at 10% laser intensity over 120 s at 2‐s intervals. The fluorescence within each cell along the filament was measured at specific time points with ImageJ; these values were then normalized to the pre‐bleach baseline to create relative fluorescence profiles. FRAP data were analyzed using GraphPad Prism software.

### Statistical analysis

All experiments were performed with at least three biological replicates. Statistical details for each experiment are provided in the figure legends. Data were analyzed and visualized using GraphPad Prism. Statistical significance was determined using a paired Student's *t*‐test, with significance levels denoted as follows: *p* < 0.05 (*), *p* < 0.01 (**), *p* < 0.001 (***), *p* < 0.0001 (****), and not significant (ns) if *p* > 0.05.

## AUTHOR CONTRIBUTIONS

**Sujuan Liu**: Data curation; formal analysis; funding acquisition; investigation; methodology; validation; visualization; writing—original draft. **Zhenyu Wang**: Investigation. **Guiming Lin**: Investigation; methodology. **Wenkai Li**: Investigation. **Xiaoli Zeng**: Writing—review and editing. **Ju‐Yuan Zhang**: Conceptualization; funding acquisition; methodology; project administration; supervision; validation; writing—original draft; writing—review and editing. **Cheng‐Cai Zhang**: Conceptualization; supervision; writing—review and editing.

## ETHICS STATEMENT

No animal or human research was involved in this study.

## CONFLICT OF INTERESTS

The authors declare no conflict of interests.

## Supporting information

Supplementary Material ‐250915.

## Data Availability

All other data supporting the findings of this study are available within the article and its supporting information materials.
