# Supplementary material for: The ribonuclease E regulator RebA is essential for diazotrophic growth in the cyanobacterium Anabaena PCC 7120
Source: mLife. 2025 Oct 27;4(5):516–26. doi: 10.1002/mlf2.70045 (PMC12575086; doi:10.1002/mlf2.70045)
Supplement: Supplementary file 1 — Supplementary Material ‐250915. [file MLF2-4-516-s001.docx]

**Supplementary Information**


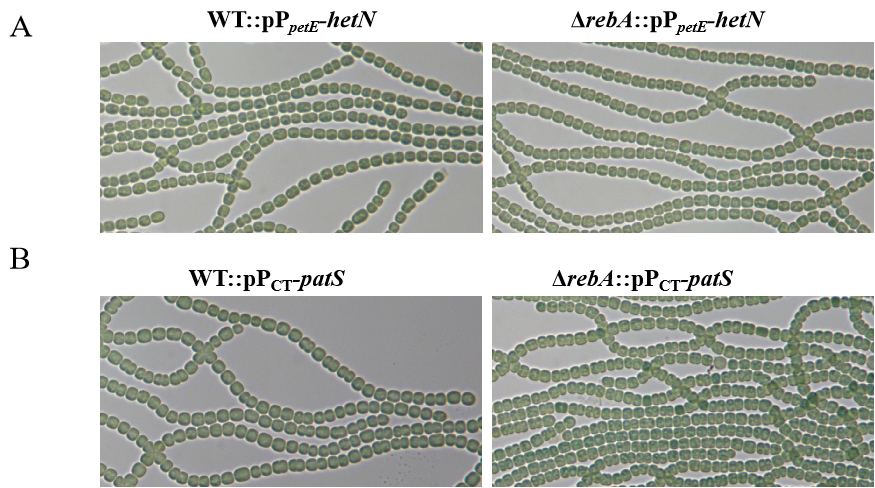


**Figure S1. Overexpression of *hetN* or *patS* suppress heterocyst formation in WT and Δ*rebA* strains. (A)** *hetN* was overexpressed using the *petE* promoter, which is inducible by Cu^2+^. Cells were pre-cultured for 3 days in the presence of 0.5 μM Cu^2+^ prior to nitrogen starvation. **(B)** *patS* was overexpressed using the CT promoter, which is inducible by Cu^2+^ and theophylline. Cells were pre-cultured for 3 days in the presence of 1.2 μM Cu^2+^ and 2 mM theophylline before nitrogen starvation. Images were captured from cultures deprived of combined nitrogen for 24 hours. Scale bar = 10 μm.

Table S1. *Anabaena* and *E. coli* strains used in this study.

| **Strains** | **Description** | **Source** |
| --- | --- | --- |
| *Anabaena* sp. PCC 7120 (WT) | the wild type strain of *Anabaena* sp. PCC 7120 | R. Haselkorn |
| Δ*rebA* | A markerless deletion mutant strain of *rebA* | 1 |
| OE-RebA | WT bearing the replicative plasmid pP*_rbcL_*-RebA; Nm^r^ | 1 |
| C-RebA | Δ*rebA* bearing pP*_rbcL_*-RebA; Nm^r^ | 1 |
| OE-RebA^V337A^ | WT bearing pP*_rbcL_*-RebA^V337A^; Nm^r^ | 1 |
| OE-RebA^l368A^ | WT bearing pP*_rbcL_*-RebA^l368A^; Nm^r^ | 1 |
| OE-RebA^I370A^ | WT bearing pP*_rbcL_*-RebA^I370A^; Nm^r^ | 1 |
| OE-RebA^V387A^ | WT bearing pP*_rbcL_*-RebA^V387A^; Nm^r^ | 1 |
| OE-RebA^I388A^ | WT bearing pP*_rbcL_*-RebA^I388A^; Nmr | 1 |
| WT::*rebA*-*gfp* | *rebA* translational fusion with *gfp* on WT chromosome; Sp^r^/Sm^r^ | This study |
| WT::pP*_hetR_*-*gfp* | WT bearing pP*_hetR_*-*gfp*; Nm^r^ | This study |
| WT::pP*_patS-_gfp* | WT bearing pP*_patS-_gfp*; Nm^r^ | This study |
| WT::pP*_hetN_*-*gfp* | WT bearing pP*_hetN_*-*gfp*; Nm^r^ | This study |
| WT::pP*_petE_*-*hetN* | WT bearing pP*_petE_*-*hetN*; Nm^r^ | This study |
| WT::pCT-*patS* | WT bearing pCT-*patS*; Nm^r^ | This study |
| Δ*rebA*::pP*_hetR_*-*gfp* | Δ*rebA* bearing pP*_hetR_*-*gfp*; Nm^r^ | This study |
| Δ*rebA*::pP*_patS-_gfp* | Δ*rebA* bearing pP*_patS-_gfp*; Nm^r^ | This study |
| Δ*rebA*::pP*_hetN_*-*gfp* | Δ*rebA* bearing pP*_hetN_*-*gfp*; Nm^r^ | This study |
| Δ*rebA*::pP*_petE_*-*hetN* | Δ*rebA* bearing pP*_petE_*-*hetN*; Nm^r^ | This study |
| Δ*rebA*::pCT-*patS* | Δ*rebA* bearing pCT-*patS*; Nm^r^ | This study |
| WT::*sepJ-gfp* | *sepJ* translational fusion with *gfp* on chromosome; Sp^r^/Sm^r^ | This study |
| Δ*rebA*::*sepJ-gfp* | Δ*rebA* with *sepJ* translational fusion with *gfp* on chromosome; Sp^r^/Sm^r^ | This study |

Abbreviations: Nm^r^, neomycin resistance; Sm^r^, streptomycin resistance; Sp^r^, spectinomycin resistance.

# Table S2. Plasmids used in this study.

| **Plasmid** | **Description** | **Source** |
| --- | --- | --- |
| pCpf1b-sp | Sp^r^/Sm^r^; CRISPR/Cpf1-based genome editing vector | 2 |
| pCpf1b-npt | Nm^r^; CRISPR/Cpf1-based genome editing vector | 2 |
| pCT | Nm^r^; a pDU1-derived vector that contains the inducible CT (Cu^2+^ and theophylline) promoter | 3 |
| pRL25T | Nmr; pDU1-based shuttle vector | 4 |
| pSYFP2 | Spr/Smr; carrying supper folding *yfp* coding sequence | 5 |
| pCpf1b-ΔrebA | Sp^r^/Sm^r^; the genome editing plasmid for *rebA* markerless deletion | 1 |
| pCpf1-All1338GFP-R1342-npt | Nm^r^; CRISPR-Cpf1 editing plasmid for *rebA* translational fusion with g*fp* | This study |
| pP*_rbcL_*-RebA | Nm^r^; pRL25N-derived plasmid for RebA expression under the relatively strong promoter P*_rbcL_* | 1 |
| pP*_rbcL_*-RebA^V337A^ | Nm^r^; pP*_rbcL_*-RebA derivate for expressing the RebA variant that has the V337A mutation | 1 |
| pP*_rbcL_*-RebA^l368A^ | Nm^r^; pP*_rbcL_*-RebA derivate for expressing the RebA variant that has the I368A mutation | 1 |
| pP*_rbcL_*-RebA^I370A^ | Nm^r^; pP*_rbcL_*-RebA derivate for expressing the RebA variant that has the I370A mutation | 1 |
| pP*_rbcL_*-RebA^V387A^ | Nm^r^; pP*_rbcL_*-RebA derivate for expressing the RebA variant that has the V387A mutation | 1 |
| pP*_rbcL_*-RebA^I388A^ | Nm^r^; pP*_rbcL_*-RebA derivate for expressing the RebA variant that has the I388A mutation | 1 |
| pP*_hetR_*-GFP | Nm^r^; pRL25N-derived plasmid for testing the transcription level of *hetR* | 6 |
| pP*_patS-_*GFP | Nm^r^; pRL25N-derived plasmid for testing the transcription level of *patS* | 6 |
| pP*_hetN_*-GFP | Nm^r^; pRL25N-derived plasmid for testing the transcription level of *hetN* | 6 |
| pP*_petE_*-*hetN* | Nmr; pRL25T carrying P*_petE_*-*hetN* fusion, used for over-expression | 6 |
| pP*_CT_*-*patS* | Nm^r^; pCT carrying *patS* under the control of the CT promoter, used for over-expression | 6 |
| pGFP-Alr2338F2256 | Sp^r^/Sm^r^; CRISPR-Cpf1 editing plasmid for *sepJ* translational fusion with *gfp* | This study |

Table S3. Oligonucleotides used in this study.

| **Name** | **Sequence (5’-3’)** | **Description** |
| --- | --- | --- |
| Palr2338F219 | tggcagaaattcgatatctagatcATCTAACTTGTCACCAGTCC | to amplify a region in *sepJ* ORF, which is used to construct the plasmid pGFP-Alr2338F2256 |
| Palr2338R2253 | accactacctccagatccaccTTCTGCATTGGCAGGTT |  |
| Palr2338F2257 | accggatcatcagtactcccACCCTTATGACAAAGGACTTATA | to amplify a region downstream *sepJ* ORF, which is used to construct the plasmid pGFP-Alr2338F2256 |
| Palr2338R3129 | cgcaacgttgttgccattgcTGACACCAAAATGCTTATGATGT |  |
| PV_16 | GGTGGATCTGGAGGTAGTGGT | to amplify a GFP region from the plasmid pSfgfp-Sp |
| Pinsert2 | GGGAGTACTGATGATCCGGT |  |
| cr_alr2338F2256F | agatAACCCTTATGACAAAGGACTTA | to generate the DNA duplex of a spacer sequence, which is used to construct the plasmid pGFP-Alr2338F2256 |
| cr_alr2338F2256R | agacTAAGTCCTTTGTCATAAGGGTT |  |
| Pall1338F428 | tggcagaaattcgatatctagatcTTAGGAGGGGACGGCAAGCCAAGCCAGCAC | to amplify a region in *rebA* ORF, which is used to construct the plasmid pCpf1-All1338GFP-R1342-npt |
| Pall1338R1269e | CACCAGAGACAAAGAATAGACCTGACCATC |  |
| Pall1338F1334a | ATCGAGATCCAAGTGATTCAACACTGT | to amplify a region downstream *rebA* ORF, which is used to construct the plasmid pCpf1-All1338GFP-R1342-npt |
| Pall1338R2239 | cgcaacgttgttgccattTGGAACCAACCCCCAGGAACTTGGTCTGAT |  |
| PGFP-Fb | gatggtcaggtctattctttgtctctggtgGGTGGATCTGGAGGTAGTGGT | to amplify a GFP region from the plasmid pSYFP2 |
| PGFP-Rb | actacctccagatccaccGGGAGTACTGATGATCCGGT |  |
| cr_all1338R1342F | agatGATCTCGATTTTATCCGTTAAT | to generate the DNA duplex of a spacer sequence, which is used to construct the plasmid pCpf1-All1338GFP-R1342-npt |
| cr_all1338R1342R | agacATTAACGGATAAAATCGAGATC |  |

**Supplementary References**

1. Liu SJ, Lin GM, Yuan YQ, Chen W, Zhang JY, Zhang CC. A conserved protein inhibitor brings under check the activity of RNase E in cyanobacteria. *Nucleic Acids Res*. 2024;52:404-19.
2. Niu TC, Lin GM, Xie LR, Wang ZQ, Xing WY, Zhang JY, et al. Expanding the potential of CRISPR-Cpf1-Based genome editing technology in the cyanobacterium *Anabaena* PCC 7120. *ACS Synth Biol*. 2019;8:170-80.
3. Zhou C, Zhang J, Hu X, Li C, Wang L, Huang Q, et al. RNase II binds to RNase E and modulates its endoribonucleolytic activity in the cyanobacterium *Anabaena* PCC 7120. *Nucleic Acids Res*. 2020;48:3922-34.
4. Yang Y, Huang XZ, Wang L, Risoul V, Zhang CC, Chen WL. Phenotypic variation caused by variation in the relative copy number of pDU1-based plasmids expressing the GAF domain of Pkn41 or Pkn42 in *Anabaena* sp. PCC 7120. *Res Microbiol*. 2013;164:127-35.
5. Zhang JY, Lin GM, Xing WY, Zhang CC. Diversity of growth patterns probed in live cyanobacterial cells using a fluorescent analog of a peptidoglycan precursor. *Front Microbiol*. 2018;9:791.
6. Wang L, Lin GM, Niu TC, Zhang SR, Zhang JY, Tang GF, et al. PatD, a gene regulated by NtcA, is involved in the optimization of heterocyst frequency in the cyanobacterium *Anabaena* sp. strain PCC 7120. *J Bacteriol*. 2019;201:e00457-19.
